# Challenges in Managing Noncommunicable Diseases: Perspectives of Family Physicians Practicing in Public‒Private Partnerships: A Qualitative Study

**DOI:** 10.1002/hsr2.70869

**Published:** 2025-06-11

**Authors:** Aboalfazl Marvi, Mohsen Aarabi, Fatemeh Kokabisaghi, Seyed Amir Soltani Kontai, Ali Vafaee‐Najar, Elaheh Hooshmand

**Affiliations:** ^1^ Student Research Committee Mashhad University of Medical Sciences Mashhad Iran; ^2^ Department of Management Sciences and Health Economics School of Health Mashhad University of Medical Sciences Mashhad Iran; ^3^ Department of Community Medicine School of Medicine Mazandaran University of Medical Sciences Sari Iran; ^4^ Social Determinants of Health Research Center Mashhad University of Medical Sciences Mashhad Iran; ^5^ Social Determinants of Health Research Center, Health Research Institute Golestan University of Medical Sciences Gorgan Iran

**Keywords:** family physician, Iran, NCDs, noncommunicable disease control, primary healthcare, public‒private partnership

## Abstract

**Background:**

Providing primary healthcare through a family physician team and referral system is an equitable and cost‐effective model for preventing and managing noncommunicable diseases (NCDs).

**Aim:**

This study aimed to explore the challenges of managing NCDs from the perspective of family physicians practicing within the public–private partnerships (PPPs) model in the urban family physician program in Mazandaran Province, a region in northern Iran.

**Methods:**

This qualitative study was conducted using a conventional content analysis approach. A group of 17 general practitioners employed through PPPs was selected through purposive sampling and the snowball method to participate in the study. Semistructured in‐depth interviews were conducted as the data collection method. Graneheim and Lundman's approach was utilized for data analysis. The coding process was performed via MAXQDA 2020 software.

**Results:**

The current challenges in managing NCDs were categorized into 8 main themes and 36 subthemes. Key themes including financing (poor financial management, lack of defined budget); equipment and infrastructure (lack of diagnostic tests and physical space); human resource (high workload, inadequate training, low motivation, and burnout); payment mechanism (issues related to per capita allocation, payment method, salary, and incentives); information system (multiple databases, poor data sharing, and low data quality); referral system (weak provider coordination, and electronic referral); health insurance (service coverage and low attention to the quality); and community engagement (weaknesses in implementing education initiatives and utilizing local capacities).

**Conclusion:**

The findings indicate significant obstacles in managing NCDs. Therefore, health policymakers and managers at the regional and national levels should develop and implement targeted plans to address key challenges in preventing and controlling NCDs through private family physician teams. Alternatively, the findings could provide insights into the private sector's participation in managing NCDs to health policymakers in other developing countries.

## Abstract (highlights)

**What Is Known in This Topic?:**

- In 2019, NCDs accounted for 74.3% of all deaths worldwide.
- The prevalence of NCDs is profoundly influenced by globalization and urbanization.
- Delivering primary healthcare through a family physician team is an effective model.
- The private sector has the potential to respond to the challenges of NCDs.

**What This Study Added?:**

- This study revealed the difficulties that private family physicians face in implementing the NCD intervention package, which can serve as important evidence for the formulation or modification of relevant policies.
- This study can present evidence for the implementation of a family physician program through private‐sector participation in Iran or other developing countries. In addition, it can provide a basis for future research.

**Abbreviations**
CHW: community health worker
EMRO: East Mediterranean Region Office
IraPEN: Iranian Package of Essential Noncommunicable Diseases Intervention
LMICs: low‐ and middle‐income countries
NCDs: noncommunicable diseases
NCDs‐GAP: Noncommunicable Diseases Global Action Plan
PFPs: private family physicians
PHC: primary healthcare
PPPs: public‒private partnerships
UFPP: urban family physician program
WHO: World Health Organization

## 1 Introduction

The prevalence of noncommunicable diseases (NCDs) poses a significant challenge to health systems [1, 2]. NCDs, including cardiovascular diseases, diabetes, cancers, chronic respiratory diseases, and their common risk factors, are the primary causes of death and disability worldwide [3]. In 2019, NCDs accounted for 74.3% of all deaths worldwide. Developing countries are disproportionately affected by these diseases. Three‐fourths of all NCD‐related deaths and approximately 86% of premature deaths (at the age of 30–69) occur in low‐income countries [1, 4]. Notably, with effective measures, 80% of these diseases can be prevented or postponed [2].

Globally, NCDs were responsible for 63.8% of disability‐adjusted life years in 2019 [1]. Due to their chronic nature, these diseases result in significant economic losses for societies, families, and patients. NCDs increase both the direct costs of healthcare and the indirect costs linked with lost productivity among the working‐age population, particularly in low‐ and middle‐income countries (LMICs) [5]. Thus, if appropriate actions are not taken, healthcare systems encounter numerous difficulties due to the economic and social impacts of NCDs [6].

According to the 2019 Global Burden of Diseases study, NCDs accounted for 83.5% of deaths and 78.1% of the disease burden in Iran [1, 7, 8]. These diseases lead to significant economic losses for the Iranian economy. A case study conducted in 2018 on the investment analysis of NCDs in Iran revealed that the economic impact of these diseases was approximately 838.49 trillion rials, which represents about 5% of the gross national product [9]. Furthermore, NCDs pose a serious challenge to Iran's healthcare system, especially in urban areas, where the primary healthcare (PHC) system lacks comprehensive services to effectively manage these diseases [10].

NCDs are multidimensional in nature, and their management requires a comprehensive and multidisciplinary approach. Adopting an all‐of‐society policy approach, especially private sector participation in the prevention and control of NCDs, is inevitable [11, 12]. The private sector has the potential to respond to the challenges of health systems [13]. Engaging in public–private partnerships (PPPs) can yield significant benefits in preventing and controlling NCDs, including improved access to funding, strengthened collaboration, high‐quality services, health promotion initiatives, innovative solutions, skill development, and more effective policies [14]. Thus, leveraging the private sector's ability to implement family physician (FP) programs in urban regions of Iran could enhance PHC's ability to address NCD issues [15].

Despite significant progress in the prevention and control of NCDs, Iran's PHC system is facing issues related to policy‐making, stewardship, resource constraints, and inadequate utilization of private sector potential [16, 17, 18]. The outsourcing of health services is a significant solution for increasing the access, justice, quality, and efficiency of health care [19]. Failing to address the obstacles and challenges of outsourcing, such as financial, accountability, and responsibility issues, can result in the program's failure and hinder the achievement of health system goals [20].

NCD patients or people at risk of these diseases need long‐term care, patient‐centered, community‐based, and sustainable interventions. Providing this care requires a health system based on PHC centered on FPs [21, 22]. FPs deliver PHC to the community in a specific region and lead the movement of the communities toward universal health coverage [23].

Due to the difficulties faced by the PHC network in addressing the needs and demands of urban residents, such as Providing quality services, and the lack of effective implementation of service packages, particularly in the prevention and control of NCDs, nutrition, and mental health, the urban family physician program (UFPP) was launched as a pilot initiative in 2012 [15, 24, 25]. This program was implemented in the two largest provinces, Fars and Mazandaran, with collaboration from both the public and private sectors. Notably, in Mazandaran Province, approximately 80% of urban family doctors are provided by the private sector [15].

Studies have shown that the UFPP faces challenges in its organizational structure, financing, payment mechanisms, rules and regulations, governance and leadership, human resources, equipment, infrastructure, information management, and monitoring and evaluation [26, 27].

To enhance the health system and meet the goal of reducing NCD mortality by 30%, Iran integrated the Package of Essential NCD Interventions (IraPEN) into its PHC system in 2016. This integration was part of a broader health sector reform plan [6]. The challenges associated with this package have not been investigated in the UFPP. In the context of NCD programs, it is crucial to understand the perspectives of private FPs to address the challenges of NCD patient care. Therefore, this study was conducted to identify the challenges of managing NCDs from the viewpoint of private FPs practicing in PPPs.

## 2 Methods

This study was conducted with qualitative content analysis methods via a conventional approach. The authors followed the consolidated criteria for the qualitative reporting research checklist (COREQ) [28] to ensure that key elements of the qualitative research were reported.

### 2.1 Study Setting and Participants

**Figure 1:**
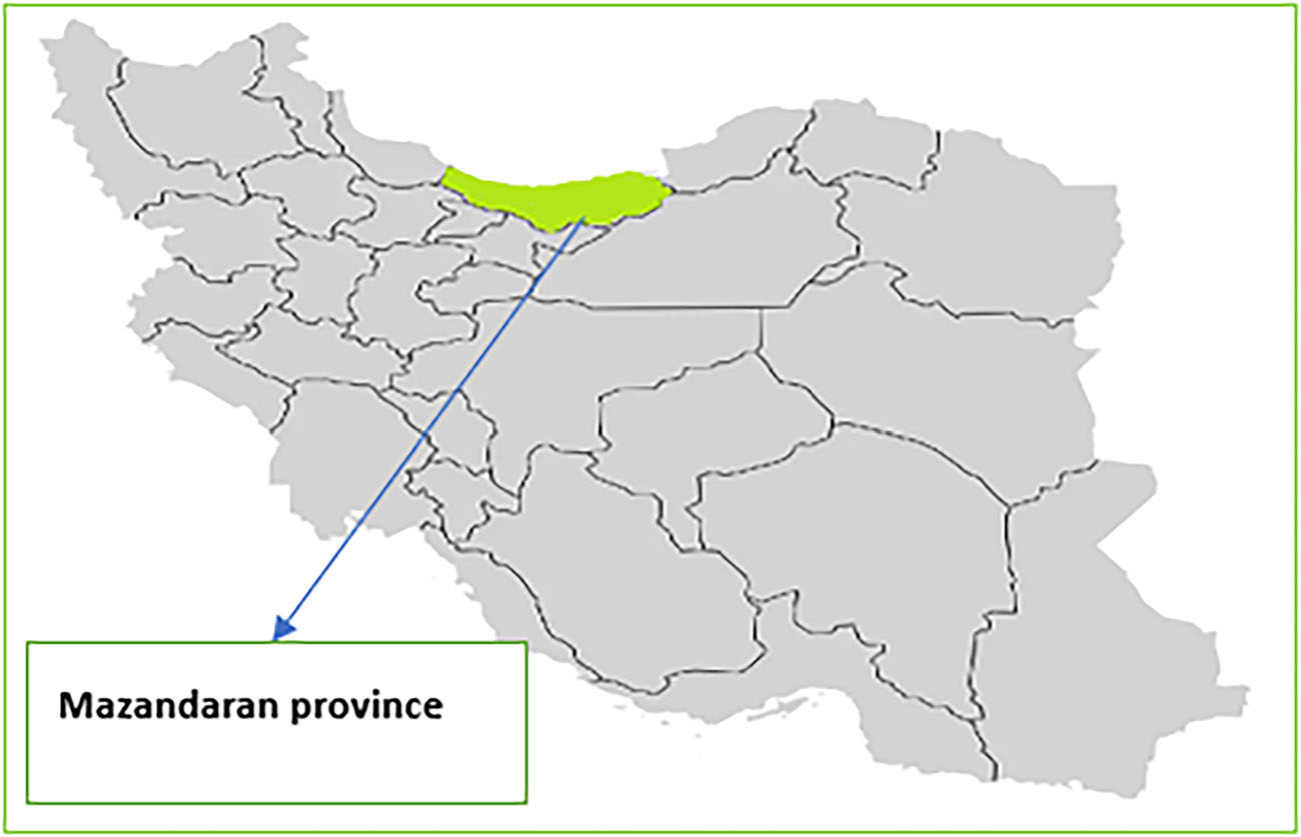
The research's location.

The study participants were general practitioners working under the PPPs model in the UFPP at Mazandaran University of Medical Sciences in northern Iran (Figure 1). These general practitioners, serving as FPs, deliver PHC to a covered population of 2500 people. Additionally, some of them were representatives of private‐sector FPs in the Mazandaran urban FPs' association or had experience in representing this association. Participants were selected through purposive sampling using the snowball method based on inclusion and exclusion criteria. To gain rich data, we selected participants with maximum diversity.

The inclusion criteria were as follows: had at least 5 years of working experience in the field of the UFPP in Mazandaran Province and were willing and interested in participation. Participants who did not want to take part or continue the interview were not eligible for the study.

### 2.2 Ethics Approval and Consent to Participate

This study was performed in accordance with the principles of the Declaration of Helsinki and approved by the Research Ethics Committee of Mashhad University of Medical Sciences (MUMS) (Code number: IR.MUMS.FHMPM.REC.1402.110). Informed consent was obtained from all study participants.

### 2.3 Data Collection

Semistructured in‐depth interviews were used as the data collection method. The data collection process lasted 2 months, from April 20, 2024, to June 20, 2024, according to the interview guide. The primary interview guide was prepared through a brief review of the literature and discussion with the research team. Following three in‐depth interviews and discussions with the research team, the interview guide was edited and developed. The first author (A. M.), a PhD student with a background in several qualitative studies in PHC and familiar with interview techniques, conducted the interviews.

A total of 17 interviews were carried out with 17 FPs. The interviews were conducted in Persian. The duration of the interviews varied between 30 and 60 min, with an average time of 55 min. Approximately 1 week before the interviews were conducted, all the participants were coordinated, and their preliminary approval was obtained via phone call. The consent form and the interview guide were subsequently sent to the participants via email, and informed consent was obtained. Additionally, the required details were provided over the phone. The interviews were held face‐to‐face in the participants' workplace (10 interviews), via phone (6 interviews), or Google Meet (1 interview), because of the interference of interview sessions with the participants' working schedules.

To protect participant privacy, face‐to‐face interviews were conducted in a quiet place with only the researcher and the participant present. During online interviews, participants were informed about the call's location and assured that no third party was present. To maintain confidentiality, participants were assured that personal information, along with audio and text files, would remain secure. Additionally, participants used headphones during telephone interviews to ensure security.

The study objectives were explained to each interviewee by the interviewer at the beginning of every interview session, followed by the research questions (see the Interview guide in Supporting Information S1) and probing questions such as “Is it possible to explain it more?” “What do you mean by this item?” or “Could you please give me an example about this item?” were asked for further clarification. The interviews were recorded with the participants' consent using a voice recorder. In addition, key points, nonverbal signs, and facial expressions were noted during each face‐to‐face interview. The interviews continued until thematic saturation was reached. After 15 interviews, no new themes emerged, but two additional interviews were conducted to confirm the saturation point.

### 2.4 Data Analysis

Following the approach of Graneheim and Lundman, the data analysis process was carried out [29]. The process of data analysis was conducted simultaneously with data collection. Therefore, after each interview, the interviewer (A. M.) and one of his colleagues (E. H.) carefully listened to the interviewees' voices at least three times and subsequently transcribed them verbatim in Word software (V 2019). Then, transcribed text and non‐verbal notes from each interview were merged and imported into MAXQDA software. Finally, the coding process was carried out with an inductive approach via qualitative analysis software (MAXQDA software version 2020).

Initially, the two researchers (A. M. and E. H.) extracted meaning units from the interview texts by reading and reviewing them at least three times and immersing themselves in the content of the interviews. The extracted units were then categorized into condensed units in the second step. In the third step, codes are generated and then grouped into subcategories based on similarities and differences in concept. In the fourth step, through the merging of subcategories with related concepts, the main themes were revealed. Moreover, the coding process was supervised by one of the authors (MA). In the end, the research team's feedback was taken into account, and necessary corrections were made.

### 2.5 Study Trustworthiness

The criteria of Lincoln and Guba were utilized to enhance study trustworthiness [30]. As a result, to ensure credibility, (a) participants were selected with maximum diversity, and (b) researchers were involved in both the research process and critical peer review. Transferability was ensured by thoroughly explaining the research process (participants, sampling methods, data collection methods, and analysis approaches) and quoting the participants' perspectives in an unbiased manner. To ensure dependability, (a) the transcribed interview content was shared with the participants for their final approval; (b) after the preliminary data analysis, the summarized findings were shared with participants to validate the themes and ensure their perspectives were accurately captured. Any inconsistencies or misinterpretations were discussed and corrected accordingly; (c) two researchers performed the coding process and dedicated sufficient time for data collection and content analysis, and a third person supervised the coding process. To ensure confirmability, (a) the researchers used member checking methods and critical review, (b) we compared the emerging themes with previous research on NCD management in PHC settings and PPPs models in Iran. This methodological triangulation helped in contextualizing our findings within broader health system challenges.

## 3 Results

The research was carried out with the participation of 17 FPs from the private sector who work in the UFPP in Mazandaran Province, Iran. The demographic characteristics of the participants are presented in Table 1.

**Table 1:** The demographic characteristics of the participants.

Variables	*N*	%
Gender		
Male	17	100
Age (year)		
41–50	10	59
51–60	7	41
Education level		
MD	17	100
Work experiences in UFPP (year)		
12	17	100
Job position		
Family physician	17	100
Representative in FPs association	8	47

**Figure 2:**
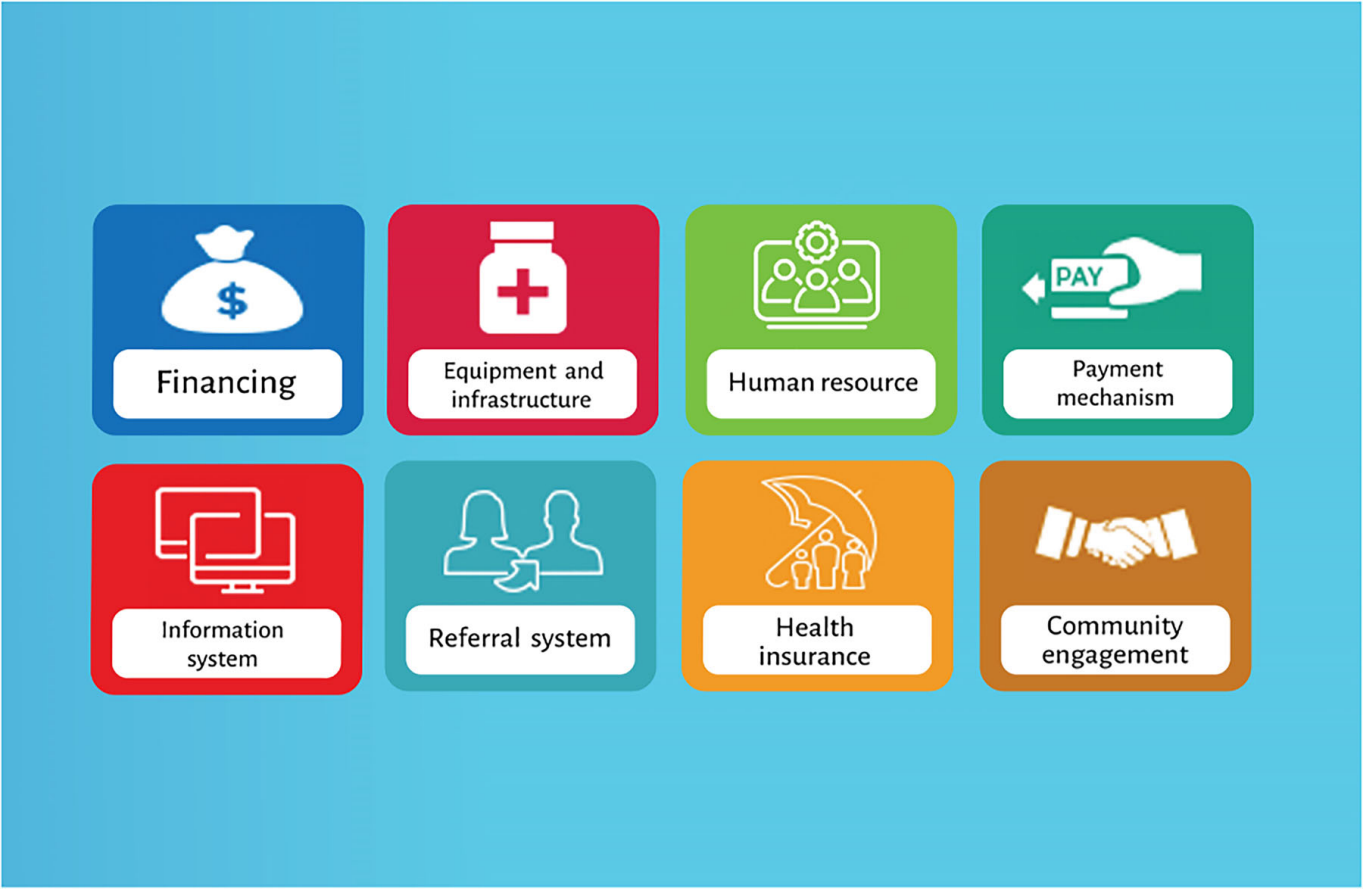
Infographic on family physicians' challenges in managing NCDs.

According to the content analysis, the challenges of managing the IraPEN as an NCD program within the UFPP from the perspective of private sector physicians were categorized into 8 themes (financing, equipment and infrastructure, human resources, payment mechanism, information system, referral system, health insurance organization, and community participation) and 36 subthemes, as shown in Table 2 and Figure 2. More details about the themes and subthemes are provided below.

**Table 2:** The challenges of managing NCDs in the urban family physician program.

Themes	Subthemes
Financing	Poor financial management in the allocation and distribution of resources
	Lack of defined funds to implement NCD programs at the PHC level
	Lower priority of preventive and community‐oriented services than specialized services in funding
Equipment and infrastructure	Insufficient availability of diagnostic tests for the screening of colorectal cancer
	Periodic shortage of HbA1C laboratory kits for the care of diabetic patients in government centers
	Insufficient laboratory centers equipped with quality technology in the public sector
	Shortage of physical space in private family physician health posts
Humane resource	Inadequacy of nonmedical workforce for existing population
	Improper retraining and insufficient skills of some staff members of family medicine teams
	Displacement of staff and a tendency for some of them to have shorter work periods
	High workload, low motivation, and burnout among some of them
Payment mechanisms	Disproportionality of per capita with the inflation rate and health posts' cost
	Inappropriate method of payment to doctors and nonmedical workforce
	Delay and irregularity of payments to family health teams
	Low salary for nonmedical staff due to low per capita allocation
	Lack of appropriate incentive items for screening, diagnosis, care, and follow‐up of NCDs
	The focus of the payments on individual care rather than community‐oriented care
Health information system	Multiple prescribing panels of insurance organizations
	Multiple databases for services delivery in the primary healthcare sector
	Lack of data sharing among of panels and databases
	Weakness of information infrastructure
	Low quality and insufficient access to data
Referral system	Poor electronic communication of databases between service delivery levels
	Weak coordination among service providers at service delivery levels
	Inadequate capacity of specialized levels in accepting referrals and providing services to patients
	Inadequate capacity of laboratory centers to accept referrals and provide services for cancer screening
	Inadequate incentives for patients and providers in referral system
	Poor performance of specialized levels in receiving, providing services, recording, and sending feedback
Health insurance organizations	Failure to update medicine coverage according to new treatment guidelines
	Inadequate coverage of laboratory, care, and follow‐up service costs
	Inadequate coverage of screening and diagnostic services for cancers
	Low attention to the quality and standard of services purchased by insurance organizations
Community engagement	Inadequacy and low continuity of educational initiatives and outreach efforts
	Weakness in implementing patients' empowerment programs (self‐care)
	Inadequate use of local capacities
	Poor cooperation of patients in regular visits

### 3.1 Financing

From the participants' perspective, the weaknesses in financial management, lack of a defined budget, and the lower prioritization of preventive services in funding allocation were the main challenges in financing. The interviewees said:

“I think, we have a fundamental problem in managing financial resources, why? Because the funds that are paid to universities of medical sciences, their managers make decisions according to the challenges and needs of the university [not necessarily the health system]. The budget of the PHC and the programs of this area, especially NCDs, should be clear. The distribution and spending of resources should also be monitored.”

(Representative of FPs, P4)

“The government should prioritize the financing of prevention and control of NCDs. For example, in the case of cancers, if we do not spend money on cancer screening, in the future, we will have to spend much more on the treatment of these diseases. The same problem applies to diabetes and high blood pressure.”

(Representative of FPs, P5)

### 3.2 Infrastructure and Equipment

The respondents highlighted several obstacles in delivering services to patients with NCDs, including insufficient physical facilities (FP health posts), poor‐quality equipment at government lab centers, and delays in the availability of screening tests like colorectal cancer (FIT Test) and HbA1c kits. In this regard:

“For colorectal cancer screening in people over 50 years of age, a test called the fit test is needed. This test should be provided to FPs, but this does not happen in practice. For example, one or two times a year, 50 or 100 tests might be delivered to the FP, which is not enough. In general, we focus more on diabetes and blood pressure than other NCDs do.”

(FP, P1)

“One of our challenges in providing care to patients, especially diabetic patients, is the lack of laboratories with up‐to‐date and high‐quality equipment. Many of our problems can be solved with quality laboratory services. Do the doctor and the patient have the right to such facilities?.”

(FP, P2)

“The HbA1c test is often not provided in government laboratories. The patient must be referred to private sector laboratories. Although they have a contract with insurance, the patient's fee is higher. This means that patients have lower access to services. In my opinion, we should either sign contracts with private laboratory centers or equip government laboratory centers.”

(FP, P9)

“One of the challenges that FPs face is the shortage of physical space to provide some healthcare services such as cancer screening.”

(Representative of PFs, P6)

### 3.3 Human Resources

The interviewees expressed concerns about the insufficient number of community health workers (CHWs), excessive workload, lack of adequate skills, poor‐quality retraining courses, and ineffective incentives, all of which contribute to dissatisfaction, burnout, staff turnover, and low retention among some doctors and other health workers. For example:

“In my opinion, the ratio of CHWs (one per 2500 people) is not enough to provide NCD‐related services or other PHC programs for the population covered. The workload of the staff is very high.”

(FP, P7)

“a private sector FP has 200 patients with hypertension and diabetes to care for. According to the instructions, these patients should be followed up monthly. The health worker must also visit and provide care to other patients. Do you think this volume of work is reasonable?”

(Representative of FPs, P3)

“I as a family doctor together with a healthcare worker work in a private health post. We work in double shifts for 8 hours a day. This has caused burnout and dissatisfaction among colleagues.”

(FP, P10)

### 3.4 Payment Mechanism

The annual increase in the per capita figure allocated for each individual, as stated by the participants, does not harmonize with the inflation rate and the expenses of private FP health posts; more importantly, this challenge has caused FPs to be unable to raise their employees' salaries rationally and pay them on time. In addition, the process and method of payment are problematic in terms of the delay in monthly payments, a focus on individual care rather than community‐oriented care (such as health education and culture building), and the lack of ineffective incentives. In this regard:

“The per capita allocation for individuals covered by an FP does not logical and fair at all. It does not meet the costs of an FP center (office rent, healthcare workers' salaries, and other expenses). This has caused dissatisfaction among FPs.”

(FP, P1)

“In addition to the fact that the allocated per capita amount is low and does not compensate for the costs and services provided, the delay in payment by insurance organizations has made work difficult. How does the FP cover office costs, staff salaries, and other costs? Interestingly, in the case of performance defects, the payment to the FP is subject to deductions, but for the delay in payments by insurance organizations, no compensation is considered.”

(PF, P12)

“Payment mechanism in the UFPP is the focus on volume versus quality in providing services. A part of the work in NCD management is community oriented and requires interventions at the community level, such as health education and culture building, which are not covered by the current payment mechanism.”

(Representative of FPs, P5)

“One of the shortcomings of the payment system is the lack of incentives for doctors and health care workers. It is difficult to provide some NCD services. Can it be done without proper incentives?”

(Representative of FPs, P6)

### 3.5 Health Information System

According to the participants, multiple electronic prescribing panels and PHC databases for registering services, and a lack of data sharing between them, are a major obstacle. In addition, the weakness of the information infrastructure, especially the Internet, along with the mentioned obstacles, has made it difficult to access quality information and, as a result, the process of providing NCD services and their continuity. The participants declared:

“These databases have not been updated according to the goals of the NCD program. One of our problems in patient care is the lack of data sharing by these systems. For example: The laboratories are not connected to the PARSA Database (a health database at the Vice Channel for Health at Mazandaran University of Medical Sciences. The patient's test results must be entered manually into the system. This process is very time‐consuming and may also cause errors in data recording.”

(FP, P11)

“One of our main challenges is the lack of integration of patients' health information systems. Currently, along with the Parsa database, new electronic prescribing systems are used by doctors. There are many electronic prescribing panels, such as Sarita, Dr. Next, and none of them are linked to the Parsa system. The fact that we do not have patient information in a comprehensive manner or rather in the form of unique electronic health records.”

(FP, P7)

### 3.6 Referral System

Lack of proper communication and cooperation between levels of service delivery, insufficient allocated incentives for patients and providers, the poor performance of medical specialists in the referral process, along with limited capacity in hospital polyclinics in accepting and providing services to NCD patients, were mentioned as important challenges of the referral system. Participants said:

“As an FP, I am responsible for the health of a group of people; specifically, if someone becomes ill and seeks my help, and if I decide to refer him to a specialist, I have to follow up until the moment the person's condition improves. Well, I have to have access to a series of information in the referral system to know what happened to my patient. For example, I refer a diabetic patient to a specialist, and the specialist provides a series of services, who are recorded in different information systems to which I do not have access. The patient comes back, and I ask about the process and results, but the patient does not know! Is it possible to provide effective care to an NCD patient?”

(FP, P4)

“In my opinion, the referral system should benefit both the patient and the service providers, especially medical specialists. Without considering incentives and meeting expectations, the referral system cannot be improved. Currently, most referrals are to the public sector and polyclinics of teaching hospitals because the private sector does not cooperate very well. The public sector is not able to provide services to a high number of patient referrals, so what happens? The quality of services decreases, and the waiting time increases.”

(Representative of FP, P5)

“The mechanism of the electronic referral system is flawed. The defined population for a FP covered by different insurance programs, such as Iranian health insurance and social security. Referral is possible electronically for patients covered by Iran Health Insurance. A more important issue is that electronic referrals are possible only in the public sector. Does the private sector not have contracts with insurance organizations? Is this an important challenge from a technical point of view? This issue hinders the quality of patient care and continuity of services too.”

(FP, P10)

### 3.7 Health Insurance Organizations

Insurance organizations face difficulties in NCD services, including weakness in adapting their coverage plans in line with the latest treatment protocols, insufficient financial support for service coverage, especially at specialized care levels, and the absence of evaluation of the quality of services provided in healthcare centers. In this regard:

“Although many efforts have been made in recent years to expand health insurance coverage in Iran, there are still many challenges. Currently, the cost of medicine and tests for patients has increased, and patients' out‐of‐pocket payments have increased.”

(FP, P7)

“Clinical guidelines for diabetes, for example, should be revised, and good quality and up‐to‐date medicines should be provided, but a major challenge we have is that the guidelines of insurance organizations are still not updated and do not cover new medicines. When I prescribe a new medicine to control a diabetic case, the patient has to pay for it. What happens is that the patient's out‐of‐pocket costs increase, and he may not be able to afford the medicine for the next visit?”

(FP, P11)

“One of the problems faced by insurance organizations is cancer screening coverage. It can be an opportunity for insurance organizations to have strategic purchases; however, we have seen less support in the past few years.”

(Representative of FP, P4)

### 3.8 Community Engagement

The individuals involved noted that there are insufficient and continuous educational programs designed and implemented for culture building, advocacy, and enhancing community engagement. They emphasized seeking support and community cooperation in the successful control of NCDs. In this context:

“One of our problems in the prevention and control of NCDs in the UFPP is the inadequacy of educational and cultural interventions. The NCD programme does not just provide care to patients with high blood pressure or diabetes. Promoting a healthy lifestyle, reducing and modifying the risk factors for NCDs, and screening target groups are among important preventive measures.”

(FP, P1)

“Society should be adequately and continuously trained, and target individuals should come to health facilities at the specified time. However, currently, most patients come to renew their medicine prescriptions, not consultations and checkups. Continuity of care for patients or other groups needs empowerment and building a culture of self‐care.”

(FP, P13)

## 4 Discussion

This qualitative research aimed to explore the obstacles encountered by private family physicians (PFPs) in managing NCDs. The finding revealed that managing NCDs through PFPs requires allocating financial resources, providing equipment and infrastructure, improving human resource management, payment mechanisms, information systems, referral mechanisms, insurance organizations' cooperation, and community involvement.

The study identified financing as a major challenge for FPs in managing NCDs, citing issues such as poor financial management, undefined funding sources, and the low prioritization of preventive and community‐based NCD services in funding allocations. In line with our findings, financing has been considered one of the main challenges of implementing NCD programs or interventions in previous studies [16, 31]. The low prioritization of funding for preventive and community‐based NCD services was a challenge. In agreement with our findings, a study in Iran indicates that the PHC sector suffers from improper financial resource allocation and distribution, due to insufficient focus on this sector compared to other health sectors [32].

According to the results, the shortages of equipment and infrastructure are some of the challenges in this field. In line with our results, previous studies have listed the lack of equipment's in health centers as one of the important obstacles to providing services to NCD patients [33, 34]. Other studies cited the lack of physical space in FP health posts to provide services, especially screening for NCDs [35, 36].

The results revealed that the disproportion of the workforce to the population, shortage of workforce, low‐quality job training, high turnover, low motivation, high workload, and burnout were among the challenges of human resource management. Studies conducted in Iran have highlighted various challenges in human resource management within PHC, including staff shortages, insufficient knowledge and skills, a lack of motivation, weaknesses in personnel supply and distribution planning, and insufficient attention to the needs and expectations of staff, especially in the workforce employed through outsourcing [17, 26]. The findings of studies in other countries have emphasized weaknesses in human resource management, such as a lack of manpower, insufficient skills, and low retention of human resources [37, 38]. Studies in India pointed out the lack of knowledge and skills of staff in service delivery [39, 40], which confirms our results.

The payment mechanism in NCD programs was criticized by study participants in terms of irrational per capita allocation, inappropriate payment methods, delays in payments, inadequate salaries for CHWs, and the lack of incentives for community care and training for the designated group.

Payment mechanisms and financial incentives to compensate for the services provided by the FP team play important roles in improving coverage, ensuring equitable access, and promoting the quality of services. Findings of studies have emphasized the necessity of designing and implementing a comprehensive and collaborative payment mechanism for health system providers [41, 42]. In line with our findings, studies in the FP domain in Iran described weaknesses in the payment system and reported similar results [26, 43].

The findings revealed that the inappropriate payment mechanism for non‐physician health workers is an obstacle to the implementation of the NCD program. One of the main challenges of Iran's PHC system in outsourcing services has been paying less attention to the quantity and quality of payments to non‐physician health workers. In addition, the shortcomings of the payment mechanism for health care workers in terms of the incompatibility of duties with the payment, delay in payments, lack of incentives, and difference between the amount received by public and private sector employees [17], which confirms our results.

According to the participants' views, the information system, in terms of a lack of data sharing between electronic prescribing panels and multiple PHC databases, along with low data quality and inadequate infrastructure, faces challenges for the care of NCDs. Findings from a research project focusing on the health systems of EMRO Region nations indicated that the information systems in these countries face organizational challenges such as having varied and fragmented databases, problems with data collection and processing, and a lack of data sharing, along with technical challenges such as equipment shortages and weak communication infrastructures [44]. Other studies reported similar results [45, 46], which confirms our findings.

The referral system faces challenges such as a lack of effective electronic communication between levels of service delivery, poor cooperation among providers, a lack of appropriate incentives for both providers and patients, poor performance of medical specialists in the referral process, and insufficient capacity in specialized healthcare centers. The referral system is one of the main components of NCD management because patients need long and continuous care throughout their lives, and this type of care requires the cooperation and coordination of other service providers [47]. An efficient referral system should be timely, safe, efficient, effective, patient‐centered, and fair; have clear instructions and specific entry criteria; and provide electronic communication between different service provision levels with a central response system [48].

Studies in Iran have shown that the electronic referral system is not performed properly due to the lack of electronic health records; the poor cooperation of service providers, particularly at specialized levels in the public and private sectors, the insufficient support of medical specialists, and the weakness in recording and sending feedback to referring centers [49, 50, 51]. The findings of studies in other countries listed the inefficient referral system as one of the obstacles in the management of NCD [52, 53]. Poor communication between service providers and recipients was cited as another challenge [40].

In addition, there were problems linked to insurance organizations, such as the failure to update the list of covered medications on the basis of recent clinical guidelines and insufficient coverage of expenses services. One study in Iran noted the weak coverage of specialized medicine for the treatment of NCDs [54]. Other studies, especially in developing countries, have reported similar results [55, 56].

Moreover, insurance organizations pay little attention to the quality and standards of services provided in health centers. Insurance organizations can play an important role in improving the quality of health services with levers such as those allocated per capita in the field of NCDs, types of payment mechanisms, and incentives. In line with our findings, the results of a systematic review in LMIC countries revealed that low attention to the quality and standards of services provided in contract health centers was one of the problems faced by insurance organizations [57].

Poor coverage of diagnostic and screening services for cancers by insurance organizations, especially in secondary‐level services, was identified as an issue in NCD management. A study in Kenya reported similar results [55].

According to the findings, the weakness of community involvement in the prevention and control of NCDs was one of the main problems. Studies in Iran have emphasized the weakness in education and cultural building programs, with a focus on the importance of preventive care and the role of FPs in providing these services [26]. Another study noted the weakness in culture building and the poor cooperation of the community in regularly visiting healthcare centers to receive basic health services related to NCDs [39, 58, 59].

The value of this qualitative study lies in its emphasis on a main public health issue that presents obstacles for health systems in most developing countries. Despite practical evidence, this study has limitations. Like other qualitative studies do, maintaining, reviewing, and demonstrating rigor is a challenge. In addition, the presence of the researchers during data collection can influence the participants' responses. Finally, this study only examined the perspectives of private‐sector FPs.

## 5 Conclusion

This study presented the views of PFPs regarding the difficulties of managing the IraPEN program at the PHC level. It was revealed that FPs working in the UFPP encounter a range of challenges in the implementation of NCD programs. These obstacles affect the performance of FPs and prevent them from achieving the goals set in NCD programs. Therefore, managing NCDs with the *participation of PFPs as an opportunity* requires the use of an effective plan and strategies. As a result, it is essential for health system policymakers and managers to implement suitable actions to address the challenges faced by NCD programs.

### 5.1 Recommendations for Future Research

The study results underscore the challenges of managing NCDs from the perspective of PFPs. Therefore, our proposal is for researchers to analyze the hurdles and strategies needed to enhance the implementation of NCD programs from the standpoint of managers and experts.

## Author Contributions

**Aboalfazl Marvi:** writing – original draft, conceptualization, writing – review and editing, software, project administration, methodology. **Mohsen Aarabi:** supervision, writing – review and editing. **Fatemeh Kokabisaghi:** writing – original draft, conceptualization, writing – review and editing, supervision, methodology. **Seyed Amir Soltani Kontai:** project administration, writing – review and editing. **Ali Vafaee‐Najar:** writing – review and editing, supervision, conceptualization, methodology. **Elaheh Hooshmand:** conceptualization, writing – review and editing, supervision, methodology.

## Conflicts of Interest

The authors declare no conflicts of interest.

## Transparency Statement

The lead author, Elaheh Hooshmand, affirms that this manuscript is an honest, accurate, and transparent account of the study being reported; that no important aspects of the study have been omitted; and that any discrepancies from the study as planned (and, if relevant, registered) have been explained.

## Supporting information

Supplemntary file 1 edited.

## Data Availability

The data that support the findings of this study are available from the corresponding author, Elaheh Hooshmand, upon reasonable request. The data are not publicly available due to their containing information that could compromise the privacy of research participants. All authors have read and approved the final version of the manuscript. Elaheh Hooshmand has full access to all of the data in this study and takes complete responsibility for the integrity of the data and the accuracy of the data analysis.
