# Supplementary material for: Challenges in Managing Noncommunicable Diseases: Perspectives of Family Physicians Practicing in Public‒Private Partnerships: A Qualitative Study
Source: Health Sci Rep. 2025 Jun 11;8(6):e70869. doi: 10.1002/hsr2.70869 (PMC12153006; doi:10.1002/hsr2.70869)
Supplement: Supplementary file 1 — Supplemntary file 1 edited. [file HSR2-8-e70869-s001.docx]

**Supplementary file 1**

**Interview guide:**

**Title**

**Challenges in Managing Noncommunicable Diseases: Perspectives of Family Physicians Practicing in Public‒Private Partnerships: A Qualitative Study**

**First part: the interview criteria**

1. The time of the interview was determined according to the participants’ working conditions and opinions.
2. The location of the interview was determined according to the participants’ working conditions and opinions.
3. The interviewer asked permission from the interviewee to record the audio file. (After explaining the objectives of the study and the ethical considerations of the research, the interviewer asked the interviewee to declare her desire to participate in the study to record it in the audio file).
4. The participants were assured that the audio file and interview texts would not be used for any other purposes under any circumstances.
5. The participants were assured that the audio file and interview texts would always remain confidential.
6. To prevent any bias, the research team provided the transcripts to the participants for confirmation after completing the interview.

**Second part: Interview questions:**

1. I would like to know your thoughts on implementing the package of essential noncommunicable disease interventions by family physicians.
2. What challenges do you face in implementing noncommunicable disease programs and interventions? What is your overall description?
3. How do you describe the family doctor team? In your view, could these programs be put into action by a doctor and a midwife or community health worker?
4. What is your opinion regarding the provision of equipment and infrastructure needed to implement noncommunicable disease services? What are the challenges?
5. How do you depict the function and position of insurance in executing noncommunicable disease programs? What do you consider to be the obstacles for insurance organizations in this area?
6. How do you describe the payment method and what obstacles does it face?
7. In your opinion, the funds needed to implement noncommunicable disease programs are provided correctly? What are the challenges in this field?
8. What is your description of electronic systems and what challenges does it have?
9. In your opinion, what are the challenges of electronic prescribing in providing services to non-communicable patients?
10. How do you characterize the referral system in the execution of non-communicable programs? Do you feel it is successful? What challenges do you face?
11. How would you define the role of society in carrying out NCD programs? What do you see as the challenges in engaging the community?
12. If possible, could you explain any topics that are not included in the questions?
13. Probing questions such as “Is it possible to explain it more?” “What do you mean by this item?”, “Could you please give me an example about that?” and … were asked in appropriate time and place by the interviewer.

Best regards,

Research team
